# Maternal-fetal attachment and perceived parental bonds of pregnant women

**DOI:** 10.1016/j.earlhumdev.2021.105310

**Published:** 2021-03

**Authors:** Kathreim Macedo da Rosa, Carolina Coelho Scholl, Lidiane Aguiar Ferreira, Jéssica Puchalski Trettim, Gabriela Kurz da Cunha, Bárbara Borges Rubin, Rayssa da Luz Martins, Janaína Vieira dos Santos Motta, Tatiane Bilhalva Fogaça, Gabriele Ghisleni, Karen Amaral Tavares Pinheiro, Ricardo Tavares Pinheiro, Luciana de Avila Quevedo, Mariana Bonati de Matos

**Affiliations:** a Postgraduate Program in Health and Behavior, Catholic University of Pelotas (UCPel), Gonçalves Chaves, 373 – 411 C, 96015-560 Pelotas, RS, Brazil; b Postgraduate Program in Epidemiology, Federal University of Pelotas (UFPel), Marechal Deodoro, 1160, 96020-220, Pelotas, RS, Brazil; c Faculty of Medicine, Federal University of Rio Grande (FURG), Avenida Itália, s/n – Km 8, Rio Grande, RS, Brazil

**Keywords:** Maternal-fetal attachment, Parental bonding, Pregnancy, Social support, Paternal role, Paternal bonding

## Abstract

**Background:**

The parental bond is characterized by the perception of care and protection received by parental figures throughout human development. During the gestational period, the intensity in which the woman manifests behaviors and feelings for the fetus was denominated maternal-fetal attachment (MFA). In this perspective, the literature indicates that there is association between MFA and the pregnant woman's perception about the bond established with her parents.

**Aims:**

This study aimed to evaluate the association between MFA and perceived parental bonds of pregnant women in the city of Pelotas/RS (Brazil).

**Study design:**

This is a cohort study with 839 women during their gestational period. All women answered to the Parental Bonding Instrument to investigate the perceived parental bonds, and the MFA was assessed through the Maternal-Fetal Attachment Scale.

**Results:**

The main results showed that perceived paternal overprotection was associated with a higher MFA after adjustment (B 2.00 CI95% 0.30; 3.70). Additionally, the pregnant women who were in the first trimester of pregnancy (*p* < 0.001), who did not live with a partner (*p* = 0.018), and who did not feel supported by the baby's father during pregnancy (*p* = 0.014) presented lower scores of MFA.

**Conclusion:**

This study showed the importance of the paternal role in the women's life, considering the perception of the bond with their father during their development, an adequate support by the father of the baby, and the presence of a partner during pregnancy. As a result, the paternal role may influence the feelings and behaviors of greater affection, care, and concern regarding the fetus.

## Highlights

• Literature suggests an association between maternal-fetal attachment (MFA) and the parental bonding of the pregnant women.
• We provide evidence that the overprotection perceived by the women with her paternal figures influences the MFA.
• The supportive behaviors provided by the baby's father influence pregnant women's MFA.
• Living with a partner also has a positive impact on MFA.

## 1 Introduction

Pregnancy is considered a period of greater emotional vulnerability in women's lives, when internal representation of the future child begins. This representation makes the pregnant women manifest, in most cases, behaviors and feelings of care, protection, and integration with the fetus. In this context, Cranley [1] defined maternal-fetal attachment (MFA) as “the extent to which women engage in behaviors that represent an affiliation and interaction with their unborn child”. It is the demonstration of care and concern over the fetus, expressed through affection, emotions, perceptions, concerns, and expectations [2].

Since the 4th month of pregnancy, the woman can feel the fetus moving inside the uterus, which makes the mother-baby interaction even more accentuated. In this period, the mother begins to have higher expectations regarding the fetus, idealizing, for instance, how their physique or personality will look like. This perception of the fetus as a human being increases the attachment that the pregnant woman has with it.

The literature shows that some factors may be associated with worse MFA, such as higher socioeconomic status, higher age, less education, lower gestational age, being primigravida, and an unplanned pregnancy. Still, other external factors that may occur before or during the gestational period may negatively influence the mother's bond with the fetus, such as previous abortions, not living with a partner and lack of social support [[3], [4], [5], [6], [7], [8], [9], [10], [11], [12], [13], [14], [15]].

Another factor that may be associated with MFA is the pregnant woman's perceived bond in relation to her parental figures during childhood and adolescence, denominated parental bond. This bond can be considered a structuring factor of personality, molding the individual's capacity of establishing relationships with other people [16]. One way to investigate this bond is through the individuals' perception of care and protection that parental figures provide them throughout their human development.

Some studies indicate that there is an association between MFA and the pregnant woman's perceived bond with her parents, in which positive memories of parental behaviors have been positively associated with MFA [[17], [18], [19]]. In the pregnancy-puerperium period, perceptions or memories of established bonds with parental figures may influence the future role of the mother and on new bonds that will be consolidated with the child. Based on this, the gestational period may contribute to the re-elaboration of her perceived relationship with her parents and to the new bonding with the fetus, which, in turn, may significantly affect the mother-child relationship and the child's development [14].

Additionally, the literature has demonstrated the negative consequences of a low MFA for the offspring. A study found that pregnant women with worse levels of MFA are more likely to have a baby with adverse neonatal results, such as prematurity and low birth weight [20]. Besides that, Punamäki et al. [20] demonstrated that a negative MFA was associated with worse language and infant sensorimotor development, as well as worse mother-baby interaction in the postpartum period. In this perspective, it is evident the importance of identifying associated factors of MFA, such as parental bond, since it is associated with negative outcomes for the mother-baby dyad.

It is important to note that studies regarding association between MFA and perceived parental bonds do not address this topic as the main subject, and usually focus on the association between MFA, anxiety, and depressive symptoms [12,14]. Considering this information and the knowledge that MFA during gestation may be influenced by memories of parental figures, this study's main objective was to evaluate the association between MFA and perceived parental bonds of pregnant women in the city of Pelotas/RS, Brazil.

## 2 Materials and methods

### 2.1 Design and participants

A population-based longitudinal quantitative study was conducted, between 2016 and 2018. The initial sample was composed of women up to 24-weeks pregnant who resided in one of the census sectors of the urban zone of Pelotas, a city in southern Brazil, that were randomly selected for the realization of the research. According to the 2010 Census of the Brazilian Institute of Geography and Statistics (IBGE), the urban zone of Pelotas contains 488 census sectors. Out of these, 244 (50%) were randomly drawn for the search of pregnant women up to the second trimester. The sample recruitment was carried out by trained health students that went from house to house in these sectors.

After identification, two evaluations were administered. In the first evaluation (baseline), a domestic interview was conducted via questionnaire containing questions about sociodemographic, pregnancy, and perceived parental bonds conditions. The second evaluation was performed in a university hospital 60 days after the first one, when the pregnant women answered a questionnaire regarding MFA and gestational age. It is worth noting that these two variables were collected at the second evaluation since the literature shows that MFA is more evident from the second gestational trimester.

We conducted a post-hoc sample power through the difference in MFA means between the groups with low care and high care, as well as between the groups of protection and overprotection, both in maternal and paternal perceived bonds found in our sample. Considering an alpha (α) of 5%, we found a power of 99.34% for a sample of 839 pregnant women.

### 2.2 Instruments

To assess MFA, the Brazilian version of the Maternal-Fetal Attachment Scale (MFAS) was used. It presented a low reliability for the Brazilian context, with a Cronbach's α of 0.63 [2]. However, the analysis for the present sample showed an α of 0.82, indicating good reliability [21]. This instrument consists of 24 questions concerning the woman's feelings and behaviors toward their fetus, evaluated through Likert-type responses ranging from 1 (never) to 5 (almost always), with item 22 being inverse-scored. The final score results from a sum of all responses, varying from 24 to 120 points. Higher scores mean a better bond between mother and fetus.

The Brazilian version of the Parental Bonding Instrument (PBI) was used to investigate the perceived parental bonds of the pregnant women. This instrument assesses, separately, the perception that the individual has of the maternal and paternal bonds during their childhood and adolescence, through 25 questions in a Likert scale, which vary from 0 (very similar) to 3 (very different). It divides parental bonding in two dimensions: perceived care and perceived overprotection [[22], [23], [24]]. This instrument was adapted and validated for Brazil, presenting a Cronbach's α of 0.91 for the dimension of care both in the maternal and paternal relationship, and a Cronbach's α of 0.87 and 0.85 for the dimension of overprotection in the maternal and paternal relationship, respectively [23]. The sum of items related to the care dimension generates a total score ranging from 0 to 36 points, and the sum of items related to the overprotection dimension generates a total score ranging from 0 to 39 points. Regarding maternal bonding, the cut-off point in the care dimension is 25, and the cut-off point in the overprotection dimension is 13. Concerning paternal bond, the cut-off points are 23 and 12, respectively [23].

The remaining variables investigated were: age (completed years), education(completed years of study), gestational age (weeks completed during pregnancy), living with a partner (no/yes), support of the father's baby (no/yes), and support of the pregnant woman's mother (no/yes). Regarding current pregnancy, the investigated variables were: first pregnancy (no/yes), prior abortions (no/yes), planned pregnancy (no/yes – responses of “no” or “kind of” were considered as “unplanned”), and fertility treatment (no/yes).

For socioeconomic status, the criteria of the Brazilian Association of Research Companies [25] was used to classify individuals into five levels (A/B/C/D/E), with A being the highest level and E being the lowest. These are based on the accumulation of material goods, education of the household head, and other characteristics such as having access to tap water and residing in a paved street. For this study, the levels were categorized and named as: higher level (A + B), middle level (C), and lower level (D + E).

### 2.3 Ethical aspects

This study is part of a larger study that was approved by the Research Ethics Committee of the Catholic University of Pelotas, under protocol CAAE 47807915.4.0000.5339. All participants received information about the study's objective and those that accepted to participate signed a Free and Informed Consent. For the pregnant women that were underage, signed authorization from a guardian was required.

### 2.4 Statistical analysis

The Statistical Package for the Social Sciences (SPSS) 22.0 software was used to run statistical analyses. Initially, a univariate analysis was conducted, with absolute and relative frequencies, mean and standard deviation. Since the dependent variable (MFA) is continuous, bivariate analyses were conducted through *t*-test, ANOVA and Pearson correlation, according to the type of the independent variable. Those variables that presented a *p*-value≤0.20 in the bivariate analysis were submitted to the multiple linear regression, considering and testing them as confounding factors [26]. It is worth highlight that the variables related with parental bonding were included in the analysis model due to presenting *p*-value≤0.20 and only then they were tested for their non-collinearity (VIF = 1.004).

The model proposed for the linear regression had three levels: sociodemographic variables (1st level); gestational and social support variables (2nd level); parental bond variables (3rd level). All variables were controlled by the same levels or previous levels, and we considered as statistically significant all associations with a *p*-value≤0,05 [26].

## 3 Results

In the first evaluation, 981 pregnant women were interviewed. Of these, 14.4% were considered losses or refusals, or lost the baby before the second evaluation. The total sample of this study involved 839 participants, who participated in the first and second evaluations.

Regarding the sample's characteristics, the mean age was M = 27.0 (Standard Deviation (SD) = 6.1) years, and the mean gestational age was M = 27.5 (SD = 6.0) weeks. Most of the women belonged to the middle socioeconomic level (57.4%), and had 11 years or more of study (57.0%). In addition, 17.9% of them did not live with a partner, 16.6% did not feel supported by their mothers concerning their pregnancy, and 6.2% did not feel supported by the baby's father regarding their pregnancy. With respect to pregnancy aspects, 57.4% were not primigravida, of which, 35.5% reporter prior abortion. Furthermore, 54.9% did not plan the pregnancy and 0.7% had undergone fertility treatment. The sample's MFA mean was M = 98.6 (SD = 11.6) points (Table 1).

**Table 1:** Sociodemographic characteristics, support, and gestational aspects of a sample of pregnant women at baseline and their association with maternal-fetal attachment after 60 days. Pelotas, Southern Brazil.

Variable	N (%)	Maternal-fetal attachment	*p*-Value
		Mean (SD)/correlation	
Age	27.0 (6.1)^a^	-0.016	0.652
Socioeconomic status			0.110
Higher	219 (26.1)	97.7 (11.3)	
Middle	482 (57.4)	99.3 (11.4)	
Lower	138 (16.4)	97.6 (12.6)	
Education			0.439
Less than 4 years	17 (2.0)	102.4 (10.4)	
Between 4 and 7 years	161 (19.2)	98.4 (12.2)	
Between 8 and 10 years	183 (21.8)	97.9 (12.1)	
11 years or more	478 (57.0)	98.8 (11.2)	
Gestational age^b^	27.5 (6.0)^a^	0.185	<0.001
Lives with a partner			0.001
No	150 (17.9)	95.7 (12.3)	
Yes	689 (82.1)	99.2 (11.3)	
Social support from baby's father			<0.001
No	52 (6.2)	93.1 (14.4)	
Yes	787 (93.8)	99.0 (11.3)	
Social support from mother			0.176
No	139 (16.6)	97.4 (12.1)	
Yes	700 (83.4)	98.8 (11.4)	
Primigravida			0.165
No	482 (57.4)	98.1 (11.7)	
Yes	357 (42.6)	99.2 (11.3)	
Prior abortion^c^			0.747
No	311 (64.5)	98.2 (11.3)	
Yes	171 (35.5)	97.9 (12.5)	
Planned pregnancy			0.067
No	461 (54.9)	97.9 (11.9)	
Yes	378 (45.1)	99.4 (11.1)	
Fertility treatment			0.330
No	833 (99.3)	98.6 (11.5)	
Yes	6 (0.7)	94.0 (17.8)	
Total	839 (100.0)	98.6 (11.6)	–

a Mean and standard deviation.

b Variable with missing data.

c The variable of prior abortion was only asked to those who were not primigravida (*n* = 482).

Regarding the association between MFA and the women's characteristics, there was a difference in attachment means concerning gestational age, living with a partner and perceiving support from the baby's father. Lower levels of attachment were observed on those who had lower gestational age (*r* = 0.185), who did not live with a partner (M = 95.7; SD = 12.3) and who reported not feeling supported by the baby's father (M = 93.1; SD = 14.4) (*p* ≤ 0.001) (Table 1).

Table 2 presents the perceived parental bonds and its association with MFA. On maternal bonding, 54.1% of the participants perceived low care, and 59.6% perceived overprotection. On paternal bonding, 44.4% of them perceived low care and 60.7% perceived overprotection. Concerning maternal bonding, lower MFA means were observed in women who perceived low care (M = 97.7 SD = 11.7; *p* = 0.016) and overprotection (M = 98.1 SD = 12.0; *p* = 0.154). With respect to paternal bonding, lower MFA means were observed in women who perceived low care and (M = 98.1 SD = 11.6; *p* = 0.370) and protection (M = 97.7 SD = 11.9; *p* = 0.104). We should mention that women who reported having no contact with a paternal figure during childhood and adolescence did not respond to this version of the instrument (*n* = 74) (Table 2).

**Table 2:** Perceived parental bond and its association with maternal-fetal attachment of pregnant women. Pelotas, Southern Brazil.

Variable	N (%)	Maternal-fetal attachment	*p*-Value
		Mean (SD)	
Maternal care			0.016
High	383 (45.9)	99.6 (11.4)	
Low	452 (54.1)	97.7 (11.7)	
Maternal overprotection			0.154
Overprotection	498 (59.6)	98.1 (12.0)	
Protection	337 (40.4)	99.3 (11.0)	
Paternal care^a^			0.370
High	426 (55.6)	98.9 (11.8)	
Low	340 (44.4)	98.1 (11.6)	
Paternal overprotection^a^			0.104
Overprotection	465 (60.7)	99.1 (11.5)	
Protection	301 (39.3)	97.7 (11.9)	
Total	835 (100.0)	98.6 (11.6)	–

a Only pregnant women that reported contact with a paternal figure during childhood and/or adolescence.

In the adjusted analysis, every increase of one gestational week increases 0.44 (95% Confidence Interval (CI) 0.31; 0.58) points in the MFAS. Women who did not live with a partner presented 2.70 less (95%CI −5.05; −0.34) points in the MFAS when compared with those who did. Moreover, women who did not feel supported by the baby's father presented 4.51 less (95%CI −8.15; −0.87) points in the MFAS when compared with those who did. As for the main exposure, women who perceived paternal overprotection presented 2.00 more (95%CI 0.30; 3.70) points in the MFAS when compared with those who perceived paternal protection. However, maternal high care (B −1.50 95%CI −3.16; 0.16) and maternal overprotection (B −0.84 95%CI −2.57; 0.88) were not associated with MFA (Table 3).

**Table 3:** Linear regression multivariate analysis considering maternal-fetal attachment and sociodemographic characteristics, support, gestational aspects, and parental bond.

	Maternal-fetal attachment		
	B	95% confidence interval	*p*-Value
1st hierarchical level			
Socioeconomic status^b^ (Higher)^a^	0.67	−0.64; 1.99	0.312

2nd hierarchical level			
Gestational age^c^	0.44	0.31; 0.58	<0.001
Lives with a partner^c^ (Yes)^a^	−2.70	−5.05; −0.34	0.025
Social support from baby's father^c^ (Yes)^a^	−4.51	−8.15; −0.87	0.015
Social support from mother^c^ (Yes)^a^	−0,97	−3.19; 1.26	0.394
Primigravida^c^ (Yes)^a^	−1.22	−2.88; 0.45	0.152
Planned pregnancy^c^ (No)^a^	1.16	−0.52; 2.83	0.175

3rd hierarchical level			
Maternal care^d^ (High)^a^	−1.50	−3.16; 0.16	0.076
Maternal overprotection^d^ (Protection)^a^	−0.84	−2.57; 0.88	0.336
Paternal overprotection^d^ (Protection)^a^	2.00	0.30; 3.70	0.021

a Reference category.

b Variable adjusted for Socioeconomic status.

c Variables adjusted for Socioeconomic status, Gestational age, Lives with a partner, Social support from baby's father, Social support from mother, Primigravida and Planned pregnancy.

d Variables adjusted for Socioeconomic status, Gestational age, Lives with a partner, Social support from baby's father, Social support from mother, Primigravida, Planned pregnancy, Maternal care, Maternal overprotection and Paternal overprotection.

## 4 Discussion

The aim of this study was to evaluate the association between MFA and perceived parental bonds of pregnant women in a city in southern Brazil. The main results showed that the pregnant women who perceived paternal overprotection during their childhood and adolescence presented better MFA. The literature on parental bonding showed that positive memories about parental practices may be associated with a better MFA. Despite this, most studies focus on the relationship of the pregnant woman to the maternal figure, and just a few studies have investigated the perception of the paternal bonding [[17], [18], [19],^27^,^28^]. Only Carvalho [17] and Van Bussel et al. [28] investigated the association between the perception of paternal overprotection and MFA. Carvalho [17] found a positive correlation between paternal overprotection and the intensity of maternal concern assessed by the Maternal Antenatal Attachment Scale (MAAS) in the second gestational trimester [29]. However, there was no association between paternal overprotection and the total score on the MAAS, concluding that paternal overprotection does not seem to significantly influence the level of maternal prenatal attachment [17]. On the other hand, Van Bussel et al. [28] found a positive correlation between paternal overprotection (assessed in the first gestational trimester) and the total MAAS score. Yet, this correlation was not maintained in the second and third trimester of pregnancy [28].

According to Parker et al. [24], overprotection is the term used to describe parental behavior characterized by control, intrusion, excessive contact, infantilization and prevention of independent behavior. This parental behavior can lead the individual to become anxious, vulnerable, fragile, dependent, and unmotivated, besides the development of mental disorders [[30], [31], [32], [33]]. However, the literature reports that an overprotective parent is also participatory, teaches boundaries, shows concern, generates security and is present to listen to the needs of the child [34]. In this perspective, despite causing damage to the psyche during development, the father's overprotective behavior may be perceived by the woman as something positive and safe, making a more positive bond to the baby by showing concern and protection.

Still, it is known that paternal behavior has vital importance for the development of the psychic structure and social organization of the child [35]. According to Vygotsky, the internal plane of the individual is not pre-existent; it is constructed by a process of internalization based on actions, social interactions, and language, and this process occurs through imitation of behaviors, especially of the parents [36]. Additionally, some authors reported that an adequate bond created by the child with his parents enables a feeling of safety to explore and obtain knowledge of the world, consequently influencing the structuring of their attachment type with other people [37,38]. In this perspective, it can be thought that women who experienced a bond of healthy concern for their father, will be better able to exercise their role as a mother in order to live together and provide positive experiences to their children, both in pre and post- childbirth [39].

It is worth mentioning that our study showed that the MFA means were high in all parental bond groups, ranging from 97.7 to 99.3, regardless of the perception. With these findings, it is evident that the woman is able to bond positively with the expected baby even though she perceives a negative bond with her parents during her development. According to Borges [39], parental experience can influence the maternal role. However, when these portraits are considered unsuccessful they end up serving as an example to be remodeled in the relationship with the child. Based on this, we can hypothesize that during the gestational period there may be a re-elaboration of the perceived relationship with the parents. Both those women who perceive a lack of affection and those who perceive overprotection do not reflect this behavior with their child and have a positive bond with them.

This study also found that pregnant women who did not live with a partner and/or did not feel supported by the baby's father regarding the current pregnancy presented less MFA means. Corroborating with our findings the literature describes that there is an association between worse MFA, not living with a partner and not feeling socially supported [3,[6], [7], [8], [9], [10],[40], [41], [42]]. Historically, taking care of the children was seen as a predominantly maternal role, while the paternal role was to financially support the family. Contrary to this understanding, men are currently more active in domestic tasks and, mainly, in following the children's development [35,43,44]. Furthermore, more recent literature reports that presence, aid and support of the partner during the gestational period are important factors that can influence the development of the maternal role and, consequently, positively interfere in MFA [[45], [46], [47]]. Complementarily, some studies describe that women who report feeling supported by the baby's father, whether they live together or not, present less symptoms of depression, anxiety and stress [48,49]. As such, the association between MFA and living with a partner as well as feeling supported by the baby's father demonstrates the relevance of a paternal figure in this important period for the woman, and the influence on feelings and behaviors of higher affection, care, and concern about the expected baby.

As expected, gestational age showed positive association with MFA. This is in accordance with the literature [1,[3], [4], [5], [6], [7], [8], [9], [10], [11], [12], [13], [14], [15],^19^]. As such, it is possible to infer that, as fetal growth occurs, the pregnant women can feel the new movements of the baby, which makes the experience more corporeal for them and may lead them to interact more adequately with the fetus.

Additionally, the limitations of this study should be considered. There is the possibility of a memory bias, since the PBI is an instrument that investigates a perception of the past. In addition, the validation of the MFAS for the Brazilian context showed an α that indicates low reliability. However, we performed the analysis on the present sample and found a Cronbach's α considered as good by the literature [21]. On the other hand, the study's design, the methodological rigor for sample selection and the sample's power should be considered as strong points of the study. In addition, few studies investigate the association between parental bonds and MFA, and some of these present methodological limitations regarding data analysis. It is also important to note that studies conducted with women, especially pregnant ones, focus on the influence of the relationship with the mother. Through the results of this study, we can emphasize the importance of the paternal role, as well as the presence of a partner and the support of the baby's father for the construction of the maternal role and the perception of a safe context so that the pregnant woman becomes more internally available to connect with the expected baby. Therefore, the need for further studies on the subject is evidenced, especially focusing on the relationship of MFA and other gestational variables, with the influence of the paternal figure.

Noting the impact and importance of more studies on the subject, studies show that worse MFA is associated with future difficulty in bonding with children and with delays in child development [8,[50], [51], [52], [53]]. Thus, the development of new intervention strategies focusing on the quality of MFA may prevent or minimize the negative consequences, especially for the children.

## Funding

This study was financed by the 10.13039/100000865Bill & Melinda Gates Foundation, the 10.13039/501100003593Conselho Nacional de Desenvolvimento Científico e Tecnológico (CNPq) and the Instituto Nacional de Ciência e Tecnologia (INCT), through protocol no. 401726/2015-0 APP/report 47/2014.

## CRediT authorship contribution statement

**Kathreim Macedo da Rosa**: Conceptualization, Formal analysis, Investigation, Writing - Original Draft, Visualization.

**Carolina Coelho Scholl**: Writing - Review & Editing, Project administration.

**Lidiane Aguiar Ferreira**: Investigation, Writing - Review & Editing.

**Jéssica Puchalski Trettim**: Writing - Review & Editing, Project administration.

**Gabriela Kurz da Cunha**: Project administration.

**Bárbara Borges Rubin**: Project administration.

**Rayssa da Luz Martins**: Investigation, Project administration.

**Janaína Vieira dos Santos Motta**: Formal analysis.

**Tatiane Bilhalva Fogaça**: Investigation.

**Gabriele Ghislen**: Supervision.

**Karen Amaral Tavares Pinheiro**: Supervision.

**Ricardo Tavares Pinheiro**: Methodology, Writing - Review & Editing, Funding acquisition.

**Luciana de Avila Quevedo**: Supervision.

**Mariana Bonati de Matos**: Formal analysis, Writing - Review & Editing, Supervision.

## Declaration of competing interest

The authors declare no conflicts of interest.
